# The Association Between Low Muscle Mass and the Risk of Depressive Symptoms: A Cross‐Sectional Study Based on the Chinese Longitudinal Health Longevity Survey (CLHLS)

**DOI:** 10.1002/brb3.70267

**Published:** 2025-02-05

**Authors:** Yuting Yang, Yan Wang, Qiao Chen, Ling Li, Wangping Jia

**Affiliations:** ^1^ Department of Medical Psychology, Daping Hospital Army Medical University Chongqing China; ^2^ Department of Wound Infection and Drug, Daping Hospital Army Medical University Chongqing China; ^3^ Department of Combat Casualty and Health Service, Daping Hospital Army Medical University Chongqing China

**Keywords:** CLHLS, depressive symptoms, exercise, low muscle mass, older adults

## Abstract

**Background:**

Many studies have shown a strong link between sarcopenia and depression, and low muscle mass (LMM) is an important component in the diagnosis of sarcopenia; however, there have been no studies on the relationship between LMM and depressive symptoms in the Chinese elderly population. To estimate the potential relationship between LMM and depressive symptoms among older adults, a cross‐sectional analysis was conducted utilizing data from the Chinese Longitudinal Health Longevity Survey (CLHLS).

**Method:**

The study sample comprised 11,711 individuals aged 65 years or older (mean age 83.0 ± 10.9) from the CLHLS database in 2018. We used the corrected appendicular skeletal muscle mass (ASM) prediction formula to assess muscle mass and the 10‐item Center for Epidemiological Studies‐Depression Scale (CES‐D‐10) to assess depressive symptoms. A multivariate logistic regression model and restricted cubic spline (RCS) curves were employed to investigate the association between LMM and depressive symptoms.

**Results:**

The study findings revealed a 1.16‐fold higher risk of depressive symptoms in the LMM group compared to the control group (adjusted odds ratio [aOR]: 1.16, 95% confidence intervals [95% CI]: 1.05–1.29, *p* < 0.001). Furthermore, for every one‐point decrease in LMM score below 7.87, the risk of depressive symptoms increased by 8%, with statistical significance. However, when the LMM score was greater than or equal to 7.87, the decrease in the LMM score did not significantly increase the risk of depressive symptoms.

**Conclusion:**

Our study suggests LMM is a risk factor for depressive symptoms in the elderly Chinese population, and within a certain range, the risk of depressive symptoms increases as the LMM score decreases. Physical exercise may be an effective strategy to maintain optimal muscle mass and help the mental health of the elderly.

## Graphical abstract

This study sought to estimate the potential relationship between low muscle mass (LMM) and depressive symptoms among older adults. Results show that LMM is a risk factor for depressive symptoms in the elderly Chinese population. Furthermore, for every one‐point decrease in LMM score below 7.87, the risk of depressive symptoms increased by 8%. However, when the LMM score was greater than or equal to 7.87, the decrease in the LMM score did not significantly increase the risk of depressive symptoms.

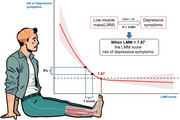

**Abbreviations:**
ASM: appendicular skeletal muscle mass
AWGS: Asian Sarcopenia Working Group;
BDNF: brain‐derived neurotrophic factor
CES‐D‐10: the 10‐item Center for Epidemiological Studies‐Depression Scale
CI: confidence interval
CLHLS: Chinese Longitudinal Health Longevity Survey
LMM: low muscle mass
OR: odds ratio
RCS: restricted cubic spline

## 1 Introduction

Depression is a prevalent mood disorder among older individuals. The incidence of depression among those aged 60 and above is approximately 5.7%, with a notable rise in frequency as age advances, peaking at 27% among individuals aged 85 and older (Devita et al. 2022; Sjöberg et al. 2017). Not only does depression lead to a decrease in the patient's quality of life, but depression also increases the risk of death by suicide, cardiovascular disease, and other diseases (Gilman et al. 2017; Péquignot et al. 2019). Depression hinders cognitive and social capabilities, resulting in reduced productivity in professional settings and other areas, thereby causing a significant financial burden on individuals, their families, and society at large (Lépine and Briley 2011). In recent decades, the global population has experienced rapid growth primarily due to the simultaneous decline in mortality and fertility rates, as well as advancements in overall quality of life. Specifically, the demographic of individuals aged 60 and above has expanded from 1 billion in 2019 to a projected 2.1 billion by 2050 (Zenebe et al. 2021). As the population expands and ages, depression has emerged as the predominant contributor to the global burden, with the societal impact of depression rising by 37.5% between 1990 and 2010 (Ferrari et al. 2013; GBD 2017 Disease and Injury Incidence and Prevalence Collaborators (2018) 2018). Effective prevention of late‐life depression can alleviate the overall economic burden and caregiving burden on society.

Multiple studies have demonstrated that physical activity is a viable intervention for depression (Schuch et al. 2016; Belvederi Murri et al. 2018; Ross et al. 2023), potentially linked to changes in brain region volume implicated in exercise‐induced depression (Gujral et al. 2017), as well as reductions in inflammation (Paolucci et al. 2018) and other factors, whereas exercise is the result of the muscular system and the skeletal system working together. Muscle mass and strength typically reach their highest levels in late adolescence or early adulthood, with a gradual decline of approximately 1%–2% in muscle mass occurring after the age of 35 (Hughes et al. 2002). Progressive loss of skeletal muscle mass and loss of muscle function are associated with increased adverse outcomes such as falls, functional decline, weakness, and death (Cruz‐Jentoft and Sayer 2019; Papadopoulou 2020). In 1989, Rosenberg introduced the term “sarcopenia” (derived from the Greek words “sarx” meaning “flesh” and “penia” meaning “loss”) to characterize the loss of muscle mass associated with aging (1989; Rosenberg 1997). According to the Asian Sarcopenia Working Group (AWGS) 2019 criteria, sarcopenia is considered when low muscle mass (LMM) is accompanied by low muscle strength or low physical function (Chen et al. 2020). Sarcopenia is identified as a driver of reduced independence and quality of life in older adults (Janssen et al. 2002), as well as an elevated susceptibility to unintentional disability and overall mortality risk (Landi et al. 2016). The prevalence of sarcopenia can reach up to 33% in the community, with even higher rates observed in long‐term care facilities and acute hospital care settings (Cruz‐Jentoft et al. 2014). In 2000, the direct costs associated with sarcopenia represented 1.5% of the total healthcare expenditures. It has been projected that a decrease of 10% in the prevalence of sarcopenia could result in savings of $1.1 billion in health‐related expenses (Janssen et al. 2004).

A study conducted on a Korean population revealed a significant correlation between low skeletal muscle mass and depressive symptoms in men, while no such association was observed in women. The findings suggest that maintaining optimal skeletal muscle mass could potentially lower the risk of developing depressive symptoms in middle‐aged Korean men (Heo et al. 2018), and there is evidence suggesting that depression may play a causal role in the development or exacerbation of muscle loss. Kahl et al.’s research demonstrated a significant impact of depression and gender on muscle mass, revealing that men with depression exhibited decreased muscle mass compared to their healthy counterparts (Kahl et al. 2017). Although the above studies highlight the close relationship between muscle mass and depression, the biochemical mechanism between muscle mass and depression is unclear and may involve multiple factors. Brain‐derived neurotrophic factor (BDNF) is an actin produced by skeletal muscle cells that crosses the blood–brain barrier (Pan et al. 1998). BDNF could affect hippocampal neurogenesis, and hippocampal volume is closely associated with depression (Campbell et al. 2004). BDNF is also a key regulator of the plasticity of various types of neurons in the brain, and studies suggest that limited plasticity may be the underlying cause of depression (Castrén and Monteggia 2021). Several studies have reported a decrease in serum and plasma BDNF levels in patients with depression and a return to baseline levels after successful treatment with antidepressants (Castrén and Monteggia 2021; Karege et al. 2002). Skeletal muscle is the main organ of glucose homeostasis, 75% of postprandial glucose intake is done by skeletal muscle, and LMM may impair glucose homeostasis, which is closely associated with depressive symptoms (DeFronzo and Tripathy 2009; Yang et al. 2022). In addition, chronic inflammation and oxidative stress may play a role in the common pathophysiological mechanisms linking sarcopenia and depression (Hashimoto et al. 2023; Bădescu et al. 2016). It may be associated with some inflammatory mediators such as C‐reaction protein, albumin, and interleukin‐1β (Morawin et al. 2023). These studies suggest that not only actin‐mediated muscle–brain endocrine circuits are involved in the association between muscle mass and depression but blood glucose metabolism, inflammation, and oxidative stress also play important roles.

Despite the above studies showing a significant correlation between muscle mass and depressive symptoms, there are still insufficient studies on the elderly in China, and the degree of muscle mass is not clearly defined for the prevention of depressive symptoms in the elderly, so it is impossible to accurately manage the mental health of the elderly group. Based on the above studies, we proposed the following hypothesis: (1) LMM may be a risk factor for depressive symptoms. (2) There may be a suitable muscle mass that is beneficial for the prevention of depressive symptoms. Therefore, we conducted this study based on data from the 2018 Chinese Longitudinal Healthy Longevity Survey (CLHLS) to determine the relationship between LMM and depressive symptoms.

## 2 Materials and Methods

### 2.1 Data Source and Analytical Sample

**FIGURE 1:**
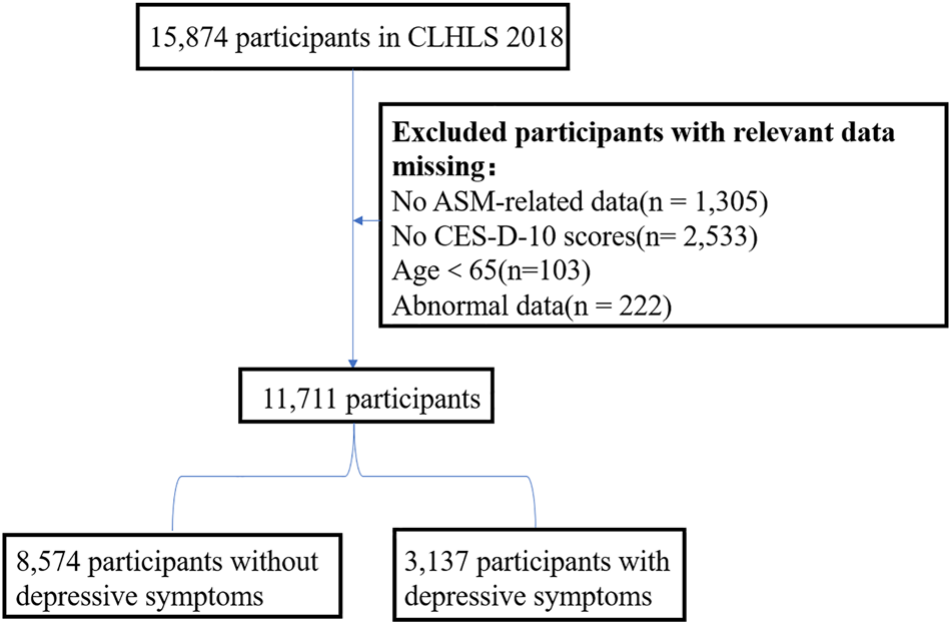
Flowchart of sampling of participants. CLHLS, Chinese Longitudinal Health Longevity Survey; CES‐D‐10, the 10‐item Center for Epidemiological Studies‐Depression Scale; ASM, appendicular skeletal muscle mass.

The cross‐sectional data for this study were obtained from CLHLS, which gathered information on health status, socioeconomic characteristics, lifestyle, quality of life, psychological attitudes, daily functioning, and accessibility of medical services among elderly Chinese individuals over a specified period (Zeng 2012). Trained interviewers gathered data via face‐to‐face surveys. To guarantee sample representativeness, a multi‐stage, stratified random cluster sampling technique was employed (Yi 2008). The survey encompassed half of China's 22 provinces and cities, effectively representing approximately 85% of the country's population. The initial baseline survey was conducted in 1998, followed by subsequent waves in 2000, 2002, 2005, 2008–2009, 2011–2012, 2014, and 2017–2018. For this study, data from the 2018 survey wave were utilized, with a cross‐sectional analysis involving 11,711 participants (Figure 1).

### 2.2 Evaluation of Depressive Symptoms and LMM

Depressive symptoms were evaluated utilizing the 10‐item Center for Epidemiological Studies‐Depression Scale (CES‐D‐10), a validated and reliable self‐reported tool for assessing depressive symptoms (Boey 1999; Zheng et al. 2023). The scale yields a total score ranging from 0 to 30, with higher scores indicating more pronounced depressive symptoms. Each item on the CES‐D‐10 scale offers four response options: “rarely,” “sometimes” (1–2 days), “occasionally” (3–4 days), and “most of the time” (5–7 days), each corresponding to numeric values from 0 to 3. A CES‐D‐10 total score of ≥ 10 is commonly regarded as indicative of the presence of depressive symptoms (Bai et al. 2023; Lei et al. 2014).

According to the AWGS 2019 consensus, leg circumference is used as a measure of muscle mass. The muscle mass was considered to be low when the circumference of the male leg was 34 cm and that of the female leg was 33 cm (Chen et al. 2020; Li et al. 2023). Details of the quality control measures are available on the CLHLS website (Center for Healthy, A., and S. Development 2020). To remove the influence of obesity type on calf circumference (Bahat 2021), we calculated appendicular skeletal muscle mass (ASM) using ASM prediction equations with reference to Ren et al. and then evaluated the LMM (Kawakami et al. 2021). The most commonly used index is ASM*height ^−2^ , adopted in the European Working Group on Sarcopenia in Older People guidelines and recommended as a skeletal muscle index (Cruz‐Jentoft et al. 2019; Kim, Jang, and Lim 2016; Cawthon et al. 2009). The ASM prediction equation was: ASM (kg) = 2.955 * sex (men = 1, women = 0) + 0.255 * weight (kg) − 0.130 * waist circumference (cm) + 0.308 * calf circumference (cm) + 0.081 * height (cm) − 11.897 (adjusted *R* ^2^ = 0.94, standard error of the estimate = 1.2 kg). The LMM was defined as < 7.0  kg*m^−2^ in men and < 5.7  kg*m^−2^ in women calculated by ASM*height^−2^ (Cruz‐Jentoft et al. 2019).

### 2.3 Potential Covariates

Based on previous studies, we collected several potential confounding variables to adjust for the associations between depression and skeletal muscle mass (Heo et al. 2018; Zhong et al. 2023). The study covariates included sociodemographic characteristics, lifestyle characteristics, and health status–related factors. Sociodemographic variables included gender, age, place of residence, marital status (married/unmarried/widowed), residency status (alone or with household), and education level (illiterate/primary school/middle school/high school or above). Lifestyle variables included smoking and drinking status, exercise, and sleep duration (< 6 h, 6–8 h, and > 8 h). Health status‐related factors included anxiety, hypertension, diabetes, cardiovascular disease, and heart disease. Exercise (yes vs. no) was assessed by asking, “Do you do exercises regularly at present?” Smoking (yes vs. no) was assessed by asking, “Do you smoke at present?” Drinking (yes vs. no) was assessed by asking, “Do you drink alcohol at present?” History of disease was based on self‐reported anxiety, hypertension, diabetes, cardiovascular disease, and heart disease.

### 2.4 Inclusion and Exclusion Criteria

The inclusion criteria for this study are as follows: (1) participants aged 65 years or older in 2018, (2) participants who answered questions on the CES‐D‐10 scale, (3) having data needed to calculate ASM. Exclusion Criteria: (1) participants aged < 65 years old; (2) missing ASM‐related data; (3) no CES‐D‐10 scores; (4) abnormal data of height, weight, waist circumference, and others. The 2018 CLHLS survey interviewed 15,874 participants; due to age < 65 years (*n* = 103), missing ASM‐related data (*n* = 1305), no CES‐D‐10 score (*n* = 2533), and abnormal data of height, weight, waist circumference and others (*n* = 222), finally, 1711 participants were selected for cross‐sectional analysis (Figure 1).

### 2.5 Statistical Analysis

We used descriptive statistics to summarize the baseline characteristics. To determine the relationship between LMM and depressive symptoms, we used multivariate logistic regression with a 95% confidence interval (CI) and developed four logistic models. The initial model did not adjust for any covariates; model 1 included socioeconomic factors (such as gender, age, place of residence, marital status, residency status, and educational level); model 2 additionally considered lifestyle factors (such as smoking, drinking, exercise, and sleep); model 3 also factored in health conditions (such as anxiety, hypertension, diabetes, cardiovascular disease, and heart disease). Restricted cubic spline (RCS) regression was used to examine a nonlinear relationship between LMM scores and depressive symptoms. All statistical analyses were conducted using R software (version 4.3.1). A bilateral test with *p* < 0.05 was considered statistically significant.

## 3 Results

### 3.1 Baseline Characteristics of Participants in 2018

A total of 11,711 individuals participated in the baseline survey of CLHLS in 2018 (Table 1). The study population was divided into two groups: 3137 participants in the depressive symptom group and 8574 participants in the control group. All covariates in Table 1 were statistically unbalanced (*p* < 0.05). The results showed that 60.6% of the participants suffered from LMM. 20.9% of participants lived alone, 3.0% were unmarried, and 51.1% were widowed. 44.5% had no educational experience. 37.2% slept less than 6 h.

**TABLE 1:** General characteristics of participants (*N* = 11,711).

Variables	Overall (*N* = 11,711)	Depressive symptoms		*p* value
		Without (*N* = 8574)	With (*N* = 3137)	
**Age (years), mean ± sd**	83.0 ± 10.9	82.6 ± 11.0	84.3 ± 10.7	< 0.001
**Weight (kg), mean ± sd**	55.8±10.3	56.3 ± 15.5	52.8 ± 16.7	< 0.001
**Height (cm), mean ± sd**	155.8 ± 10.3	156.5 ± 10.2	153.7 ± 10.4	< 0.001
**Waist circumference (cm), mean ± sd**	85.0 ± 11.4	85.7 ± 11.3	83.1 ± 11.6	< 0.001
**Calf circumference (cm), mean ± sd**	31.6 ± 6.0	31.8 ± 5.9	31.1 ± 6.4	< 0.001
**LMM_score, mean ± sd**	6.0 ± 1.9	6.1 ± 1.9	5.8 ± 2.0	< 0.001
**LMM, *n* (%)**				< 0.001
No	4609 (39.4%)	3551 (41.4%)	1058 (33.7%)	
Yes	7102 (60.6%)	5023 (58.6%)	2079 (66.3%)	
**Living status, *n* (%)**				< 0.001
With household	9260 (79.1%)	6963 (81.2%)	2297 (73.2%)	
Alone	2451 (20.9%)	1611 (18.8%)	840 (26.8%)	
**Marital status**				< 0.001
Married	5370 (45.9%)	4185 (48.8%)	1185 (37.8%)	
Unmarried	353 (3.0%)	242 (2.8%)	111 (3.5%)	
Widowed	5988 (51.1%)	4147 (48.4%)	1841 (58.7%)	
**Education level**				< 0.001
Illiterate	5209 (44.5%)	3475 (40.5%)	1734 (55.3%)	
Primary school	4066 (34.7%)	3132 (36.5%)	934 (29.8%)	
Middle school	1265 (10.8%)	1031 (12.0%)	234 (7.5%)	
High school or above	1171 (10.0%)	936 (10.9%)	235 (7.5%)	
**Sleeping duration (h)**				< 0.001
< 6	4362 (37.2%)	2672 (31.2%)	1690 (53.9%)	
6–8	4362 (37.2%)	3511 (40.9%)	851 (27.1%)	
> 8	2987 (25.5%)	2391 (27.9%)	596 (19.0%)	

Abbreviation: LMM, low muscle mass.

### 3.2 Association Between Depressive Symptoms and LMM

To assess the association between depressive symptoms and LMM, we constructed four models using multivariate logistic regression analysis (Table 2). As a binary variable, the model 3 indicated that the risk of depressive symptoms was 1.16 times higher in the group with LMM compared to the control group (odds ratio [OR]: 1.16, 95% CI: 1.05–1.29, *p* < 0.001). The results were consistent across models [OR = 1.13 for Model 1, OR = 1.12 for Model 2, OR = 1.39 for preliminary model, all *p* < 0.001].

**TABLE 2:** Multivariate logistic regression models on LMM and depressive symptoms.

LMM	Preliminary model		Model 1		Model 2		Model 3	
	OR (95% CI)	*p* value	OR (95% CI)	*p* value	OR (95% CI)	*p* value	OR (95% CI)	*p* value
No	1		1		1		1	
Yes	1.39 (1.28, 1.51)	< 0.001	1.13 (1.03, 1.24)	< 0.001	1.12 (1.01, 1.23)	< 0.001	1.16 (1.05, 1.29)	< 0.001

Abbreviations: 95% CI, 95% confidence intervals; LMM, low muscle mass; OR, odds ratio.

Preliminary model: No covariates were adjusted.

Model 1: Corrects for socioeconomic characteristics (gender, age, place of residence, marital status, residency status, education level).

Model 2: Further correction of lifestyle habits (smoking, drinking, exercise, sleeping).

Model 3: Further correction for health conditions (anxiety, hypertension, diabetes, cardiovascular disease, heart disease).

### 3.3 RCS Regression Between Depressive Symptoms and LMM

**FIGURE 2:**
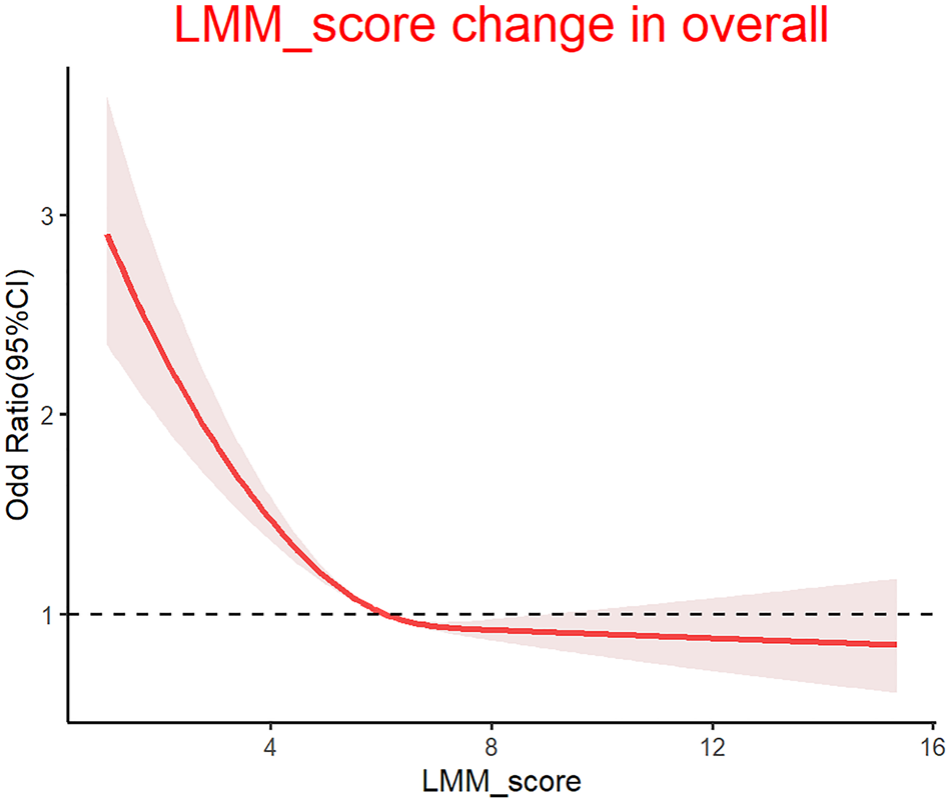
The nonlinear relationship between LMM score and depressive symptoms. LMM, low muscle mass; OR, odds ratio; 95% CI, 95% confidence intervals.

To further investigate the relationship between LMM and the risk of developing depressive symptoms, we conducted RCS regression analyses. As shown in Figure 2A, there was a significant nonlinear correlation between the LMM score and the risk of depressive symptoms, and the nonlinear correlation was “L”‐shaped. Further determine the cutoff value: 7.876864. The results showed that when the LMM < 7.87, the risk of depressive symptoms increased by 8% for every one‐point decrease in the LMM score, and the difference was statistically significant. When the LMM was greater than or equal to 7.87, the decrease in LMM score did not significantly increase the risk of depressive symptoms. Table 3 presents that when LMM< 7.87, the LMM score is negatively correlated with depressive symptoms, while when LMM≥7.87, the LMM score is not significantly correlated with depressive symptoms.

**TABLE 3:** Association between LMM score and risk of depressive symptoms.

	LMM < 7.87		LMM ≥ 7.87	
	OR	95% CI	OR	95% CI
Preliminary model	0.83	(0.81, 0.86)	1.03	(0.99, 1.07)
Model 1	0.92	(0.88, 0.96)	1.00	(0.97, 1.05)
Model 2	0.92	(0.88, 0.97)	1.01	(0.96, 1.05)
Model 3	0.92	(0.88, 0.97)	1.00	(0.96, 1.04)

Abbreviations: 95%CI, 95% confidence intervals.; LMM, low muscle mass; OR, odds ratio.

Preliminary model: No covariates were adjusted.

Model 1: Corrects for socioeconomic characteristics (gender, age, place of residence, marital status, residency status, education level).

Model 2: Further correction of lifestyle habits (smoking, drinking, exercise, sleep).

Model 3: Further correction for health conditions (anxiety, hypertension, diabetes, cardiovascular disease, heart disease).

## 4 Discussion

In this research, individuals who were 65 years old or above were chosen from the 2018 CLHLS database. The natural process of aging typically involves a gradual decline in muscle mass of around 1% after the age of 30, with this decline accelerating after reaching 70 years of age (Kim and Choi 2013). Depression is a prevalent mood disorder in older adults, with rates as high as 49% in community or nursing home settings (Devita et al. 2022). Some previous studies have assessed the relationship between these two variables, and this study is the first to investigate the association between muscle mass and depressive symptoms in the Chinese elderly using data from the CLHLS. The results of our study revealed that individuals with LMM had a higher likelihood of developing depressive symptoms. A previous study by Gao et al. also demonstrated that individuals with sarcopenia had a 49% higher chance of developing new‐onset depressive symptoms compared to those without sarcopenia, even after adjusting for potential confounding variables (Gao et al. 2021). Another study found depressed men showing a decrease in muscle mass, while no such association was observed in women (Kahl et al. 2017). Zhong et al. further demonstrated a linear relationship between depression and sarcopenia (Zhong et al. 2023). These results suggest that muscle mass is closely related to depressive symptoms, and evaluating muscle mass in the community may be a simple and effective way to identify depressive symptoms early. However, while previous studies have indicated that muscle loss may increase the risk of depression and that improving muscle quality may reduce this risk (Heo et al. 2018), none have specified the specific level of muscle quality that needs to be maintained to lower the risk of depression. Our study observed that when the LMM was below 7.87, the risk of experiencing depressive symptoms increased as the LMM score decreased. Conversely, when the LMM was equal to or greater than 7.87, a decrease in the LMM score did not significantly elevate the risk of depressive symptoms. Our study is the first to show that, at a range, depressive symptoms are negatively associated with LMM scores. Sarcopenia is associated with malnutrition (Delibaş et al. 2021), physical inactivity (Steffl et al. 2017), frailty, decreased mobility, and so on, and these changes can directly affect depression (Gu 2022; Soysal et al. 2017). The above studies confirm that sarcopenia and depression are closely related, and they may share pathophysiological mechanisms, which include inflammation (Kiecolt‐Glaser, Derry, and Fagundes 2015; Fulop et al. 2017), mitochondrial dysfunction (Bansal and Kuhad 2016; Kim et al. 2023), and vitamin D deficiency (Remelli et al. 2019; Anglin et al. 2013). Furthermore, the coexistence of sarcopenia and fat accumulation can lead to the development of a condition known as “sarcopenic obesity.” Studies have shown that sarcopenia and obesity form a vicious circle in the body, resulting in the potential for insulin resistance, mitochondrial dysfunction, increased inflammation, and other related health issues (He et al. 2021). A longitudinal investigation demonstrated that a rise in moderate to vigorous physical activity was linked to a 36% reduction in the occurrence of sarcopenia over 5 years (Mijnarends et al. 2016). Engaging in physical activity has been shown to promote the growth of muscle tissue, decrease body fat, and improve muscle strength, endurance, immune response, and cardiovascular health (Thyfault and Bergouignan 2020; Distefano and Goodpaster 2018; Belanger, Rao, and Robbins 2022). It is considered a crucial component of interventions aimed at addressing age‐related sarcopenia (Distefano and Goodpaster 2018). Aerobic exercise has been found to be effective in addressing mitochondrial dysfunction, while resistance training can help increase muscle mass and improve muscle function (Yoo et al. 2018). In the case of elderly individuals diagnosed with sarcopenia, there exist substantial or moderate levels of certainty regarding the efficacy of resistance training, either in isolation or in conjunction with nutritional support, as well as the combined approach of resistance training with aerobic exercise and balance training, in enhancing the overall quality of life. Incorporating nutritional strategies alongside exercise regimens was found to yield a more pronounced impact on grip strength compared to exercise alone while demonstrating comparable effects on various other indicators of physical function (Shen et al. 2023). Physical exercise has been shown to be an effective method for treating severe depression in adults. Furthermore, it appears that late‐life depression also responds positively to exercise. Individuals with depression can experience pleasure and emotional enhancement through engaging in physical activity (Belvederi Murri et al. 2018). The therapeutic effects of exercise on depression are attributed to a combination of psychological and physiological mechanisms. Psychological mechanisms encompass psychosocial and cognitive factors, whereas physiological mechanisms involve anti‐inflammatory properties, neuroplasticity, and various biochemical factors (Xie et al. 2021). A review article suggests that the prefrontal cortex, anterior cingulate cortex, hippocampus, and corpus callosum are important neuroanatomical markers that overlap between structural abnormalities in the brain in depression and the effects of exercise on adult brain structure. These brain regions can serve as targets for exercise therapy in treating depression (Gujral et al. 2017). Therefore, physical activity has been shown to have a dual benefit in addressing depressive symptoms, as it can directly alleviate symptoms and also indirectly combat depression by enhancing muscle mass and function. Increasing levels of physical activity could serve as a viable strategy for enhancing the mental well‐being of elderly people in the community.

### 4.1 Limitations and Advantages of This Study

First and foremost, it is important to acknowledge that the generalizability of our findings may be restricted due to the characteristics of our study population. Participants in our study were required to be active enough to participate in a voluntary health survey, which means that individuals who were bedridden or hospitalized were not included. This limitation could potentially impact the overall applicability of our results to a broader population. Second, the use of the CES‐D‐10 as a measure of mental health in our study should be noted. While the CES‐D‐10 is a commonly used tool for assessing depressive symptoms and has demonstrated good reliability, it is important to recognize that it is based on the self‐reported feelings of participants rather than a clinical diagnosis made by a healthcare professional. This reliance on subjective self‐reporting may introduce the possibility of false positives and should be taken into consideration when interpreting the results of our mental health screening. Muscle mass was estimated using previously validated anthropometric equations and not bioimpedance analysis or dual x‐ray absorptiometry as recommended by AWGS (Chen et al. 2014). While these equations have been validated and are commonly used in research settings, it is important to recognize that they may not provide the most accurate measurement of muscle mass (Cruz‐Jentoft et al. 2010). Third, we analyzed the relationship between LMM and depressive symptoms in older adults based on cross‐sectional studies rather than cohort studies. Therefore, the associations we observe may not be causal. Fourth, due to the lack of CES‐D‐10 scale and ASM‐related data, as well as a few abnormal data, 4163 participants were excluded. Exclusion of participants may result in a selection bias in the results. Finally, this study used observational data that may have biased the observed relationship by introducing confounders; although we adjusted the analysis of many potential confounders, we were unable to eliminate the possibility of residual confounding, for example, nutritional status, hypoalbuminemia, eating habits, etc. Still, our discoveries are significant. Our study further confirms the close relationship between depressive symptoms and muscle mass. The negative correlation between LMM and depressive symptoms within a range could provide valuable insights into the prevention and treatment of depression and point to the potentially positive impact that maintaining appropriate muscle mass could have on mental health in older adults. This highlights the importance of using exercise as a key strategy to prevent and manage the mental health of older adults in the community. Subsequent studies could enhance cohort research to establish the cause‐and‐effect relationship between LMM and depressive symptoms, as well as examine the impact of increased physical activity and enhanced muscle mass on individuals experiencing depressive symptoms. Furthermore, it is crucial to explore the underlying mechanisms that elucidate associations between muscle mass and depression to guide the development of interventions aimed at alleviating the financial and medical burden on individuals suffering from depression.

## 5 Conclusion

Our study suggests LMM is a risk factor for depressive symptoms in the elderly Chinese population, and within a certain range, the risk of depressive symptoms increases as the LMM score decreases. Physical exercise may be an effective strategy to maintain optimal muscle mass and help the mental health of the elderly.

## Author Contributions

Y.T. contributed to the study design and data analysis and interpretation and drafted the manuscript. Y.W. contributed to the data analysis and interpretation and critical revision of the manuscript. Q.C. contributed to the administration of the survey and critical revision of the manuscript. W.P. and L.L. contributed to the design of this study, interpretation of data, and critical revision of the manuscript. W.P. has the primary responsibility for the final content. All authors read and approved the final manuscript.

## Conflicts of Interest

The authors declare no conflicts of interest.

### Peer Review

The peer review history for this article is available at https://publons.com/publon/10.1002/brb3.70267

## Data Availability

The survey data are available from the Peking University Open Research Data Platform (Website: https://doi.org/10.18170/DVN/WBO7LK) and the data manager (nc.ude.ukp.dsn@sdahc) for researchers who meet the criteria for data access.
